# Safety and efficacy of lecanemab for Alzheimer's disease: a systematic review and meta-analysis of randomized clinical trials

**DOI:** 10.3389/fnagi.2023.1169499

**Published:** 2023-05-05

**Authors:** Yue Qiao, Yuewei Chi, Qingyuan Zhang, Ying Ma

**Affiliations:** Department of Neurology, Shengjing Hospital of China Medical University, Shenyang, China

**Keywords:** Alzheimer's disease, BAN2401, meta-analysis, lecanemab, cognitive function

## Abstract

**Objective:**

We performed a systematic review and meta-analysis of the cognitive effectiveness and safety of lecanemab on subjects with Alzheimer's disease (AD).

**Methods:**

We screened the literature published before February 2023 in PubMed, Embase, Web of Science, and Cochrane that were searched for randomized controlled trials testing lecanemab for the treatment of cognitive decline in patients with MCI or AD. Outcomes measured were CDR Sum of Boxes (CDR-SB), Alzheimer's Disease Composite Score (ADCOMS), AD Assessment Scale–Cognitive Subscale (ADAS-Cog), Clinical Dementia Rating (CDR), amyloid PET Standardized Uptake Volume Ratio (SUVr), amyloid burden on PET, and risks for adverse events.

**Results:**

A total of four randomized controlled trials were included, involving 3,108 AD patients (1,695 lecanemab groups and 1,413 placebo groups) to synthesize evidence. Baseline characteristics of the two groups were similar in all outcomes except that ApoE 4 status and higher MMSE score were observed in the lecanemab group. It is reported that lecanemab was beneficial to stabilize or slow down the decrease in CDR-SB (WMD: −0.45; 95% CI: −0.64, −0.25; *p* < 0.00001), ADCOMS (WMD: −0.05; 95% CI: −0.07, −0.03; *p* < 0.00001), ADAS-cog (WMD: −1.11; 95% CI: −1.64, −0.57; *p* < 0.0001), amyloid PET SUVr (WMD: −0.15; 95% CI: −0.48, 0.19; *p* = 0.38), amyloid burden on PET (WMD:−35.44; 95% CI: −65.22,−5.67; *p* = 0.02), adverse events (subjects with any TEAE) (OR: 0.73; 95% CI: 0.25, 2.15; *p* = 0.57), ARIA-E (OR:8.95; 95% CI: 5.36, 14.95; *p* < 0.00001), and ARIA-H (OR:2.00; 95% CI: 1.53, 2.62; *p* < 0.00001) in early AD patients.

**Conclusion:**

Our analysis found that lecanemab showed significant positive statistical efficacy with respect to cognition, function, and behavior in patients with early AD though the actual clinical significance is yet to be established

**Systematic review registration:**

https://www.crd.york.ac.uk/PROSPERO/#recordDetails, identifier: CRD42023393393.

## 1. Introduction

Alzheimer's disease (AD) is a neurodegenerative disease, which is characterized by a chronic or progressive deterioration in cognitive function, affecting more than 50 million people around the world. It was officially listed as the seventh leading cause of death in 2020 and 2021, and COVID-19 entered this category according to a study in 2022 (Tiwari et al., 2019). Alzheimer's disease is the main cause of dementia, and nearly 60% to 80% of cases will be upgraded. Drugs approved by the Food and Drug Administration (FDA) for the treatment of AD, including acetylcholinesterase inhibitors, donepezil, rivastigmine, and galanthamine, can only improve the symptoms of AD patients but cannot slow down or reverse the progress of the disease, resulting in a large number of unmet medical needs (Ayaz et al., 2015). Although today the diagnosis of AD has been developed into maturity with high accuracy, including molecular imaging approaches such as tau PET, amyloid PET, or 18F-FDG PET, no effective disease-modifying treatment is yet available (Wilson et al., 2019). The search for a drug that is able to positively influence the course of AD is a long story of frustration. Recently, investigators of lecanemab trials reported some relatively promising effects, therefore, re-energizing the popularity of the amyloid hypothesis and the interest in monoclonal antibodies against Aβ.

The abnormal brain accumulation of amyloid-beta (Aβ) proteins and intraneuronal neurofibrillary tangles of tau proteins, as well as neuroinflammation, are the key pathogenic events in the development and progression of AD (Ising et al., 2019). Reducing the levels of Aβ aggregation and plaques or increasing the brain clearance rate of Aβ is considered an effective therapeutic target for AD (Cummings et al., 2019). It is generally believed that the imbalance of amyloid β in generation and clearance is an initiating factor in the development of AD (Tublin et al., 2019). Monoclonal antibodies (mAbs) are capable of inhibiting the pathologic amyloid β oligomers or plaque formation, which has received considerable attention in slowing the progression of AD (Shankar et al., 2008). For instance, aducanumab has been demonstrated to delay the clinical progression of AD, which has selectivity for fibrillary amyloid results in support of the amyloid hypothesis (Sevigny et al., 2016). Recently, several specific treatments for AD are now being developed in patients with early AD, people at a preclinical stage of familial AD, and asymptomatic individuals at high risk of AD (Panza et al., 2019). In a recent study, mAb 158 has a unique selectivity for soluble Aβ fibrils. Lecanemab (BAN 2401) is a humanized monoclonal antibody of IgG 1, which is derived from mouse precursor MAB 158 (Englund et al., 2007; Sehldin et al., 2016). Lecanemab has a higher selectivity for protofibrils (>1000-fold) and better binding to protofibrils than to fibrils (10- to 15-fold) (Lord et al., 2009; Sehlin et al., 2012). Some recent preclinical studies have shown monoclonal antibodies (mAbs) rescue neurons from Aβ-induced cell death, which is associated with improvements in brain perfusion and neuronal viability, supporting the view that lecanemab may be an effective agent in the treatment of AD (Nikitidou et al., 2017; Sollvander et al., 2018; Gustavsson et al., 2020; Tam et al., 2021).

After aducanumab, lecanemab is the second FDA-approved anti-amyloid drug to treat AD, purportedly to be the only one that shows a statistical benefit (Barthel, 2023). This year, lecanemab was marketed with accelerated approval in the United States, and the first patient infusion was completed (100 mg/kg IV infusion). This FDA approval is based on positive results of phase 2 b proof-of-concept clinical trial in early Alzheimer's disease, and lecanemab completely removed Aβ plaques, alleviated cognitive decline, and had a low incidence amyloid-related imaging abnormalities (ARIA) in early AD (Swanson et al., 2021). In addition, the safety and efficacy of lecanemab have been investigated in a phase 3 trial (clarity AD) in participants with early Alzheimer's disease (Van Dyck et al., 2023). However, at present, there is no systematic review or meta-analysis comparing the efficacy and safety of lecanemab in the treatment of AD. With this systematic review and meta-analysis, we aimed to report a pooled analysis and evidence of monoclonal antibodies lecanemab on clinical outcomes of cognitive function, biomarkers related to Aβ and tau pathologies, and the risk for adverse events associated with this drug class and ARIA in patients with AD.

## 2. Materials and methods

### 2.1. Literature search

This evidence-based analysis is based on the preferred reporting item for systematic review and meta-analysis (PRISMA) 2020 statement (Page et al., 2021) and is expected to be registered in the PROSPERO (CRD 42023393393). By February 2023, the two authors independently identified the studies published in English through title and abstract in PubMed, Embase, Web of Science, and Cochrane which compared the efficacy and/or safety between lecanemab and placebo for the treatment of AD. We used the keywords “lecanemab”, “lecanemab-irmb”, “leqembi”, “BAN 2401”, and “Alzheimer's disease” and combinations of them (Supplementary Table 1). In addition, a list of references for all eligible studies was manually reviewed. In total, two researchers independently searched for and evaluated the included studies. Any disagreement in the literature search was resolved by consensus.

### 2.2. Identification of eligible studies

In order to be included in the systematic review, a study must have met the following selection criteria: (1) the study design being randomized controlled, comparing lecanemab and placebo; (2) assessing patients with a diagnosis of early AD (amyloid positive) with Clinical Dementia Rating (CDR) global score of 0.5 or 1 according to a clinical subgroup (mild cognitive impairment due to Alzheimer's disease or mild Alzheimer's disease-related dementia); and (3) sufficient data to calculate ratio (OR) or weighted average difference (WMD).

We excluded comments, letters, editorial comments, case reports, conference abstracts, unpublished articles, cell or animal research, and non-English articles. In addition, studies that failed to report cognitive/functional results were excluded.

### 2.3. Data extraction

Data extraction is independently completed by two researchers from the confirmed articles and collected in an Excel spreadsheet. Any differences are resolved by the third researcher to make a final determination. We collected the following data from each study: first author, publication year, registration of RCTs, study period, country of study, study design, sample size, age, AD Assessment Scale–Cognitive Subscale (ADAS-Cog), Mini-Mental State Examination (MMSE), clinical subgroup, Clinical Dementia Rating (CDR), CDR Sum of Boxes (CDR-SB), amyloid PET Standardized Uptake Volume Ratio (SUVr), amyloid burden on PET, Alzheimer's Disease Composite Score (ADCOMS), adverse events (subjects with any TEAE), amyloid-related imaging abnormalities with edema or effusions (ARIA-E) or with cerebral microhemorrhages, cerebral macrohemorrhages (ARIA-H), and risks for adverse events. When continuous variables in the study are reported as having an extreme range, quartile deviation, or median value of the data extracted from statistical charts, we calculated the mean ± standard deviation through the validated mathematical method (Hozo et al., 2005; Wan et al., 2014). When data in the study are missing or not reported, we contacted the corresponding authors to obtain complete data.

### 2.4. Quality assessment

**Figure 1:**
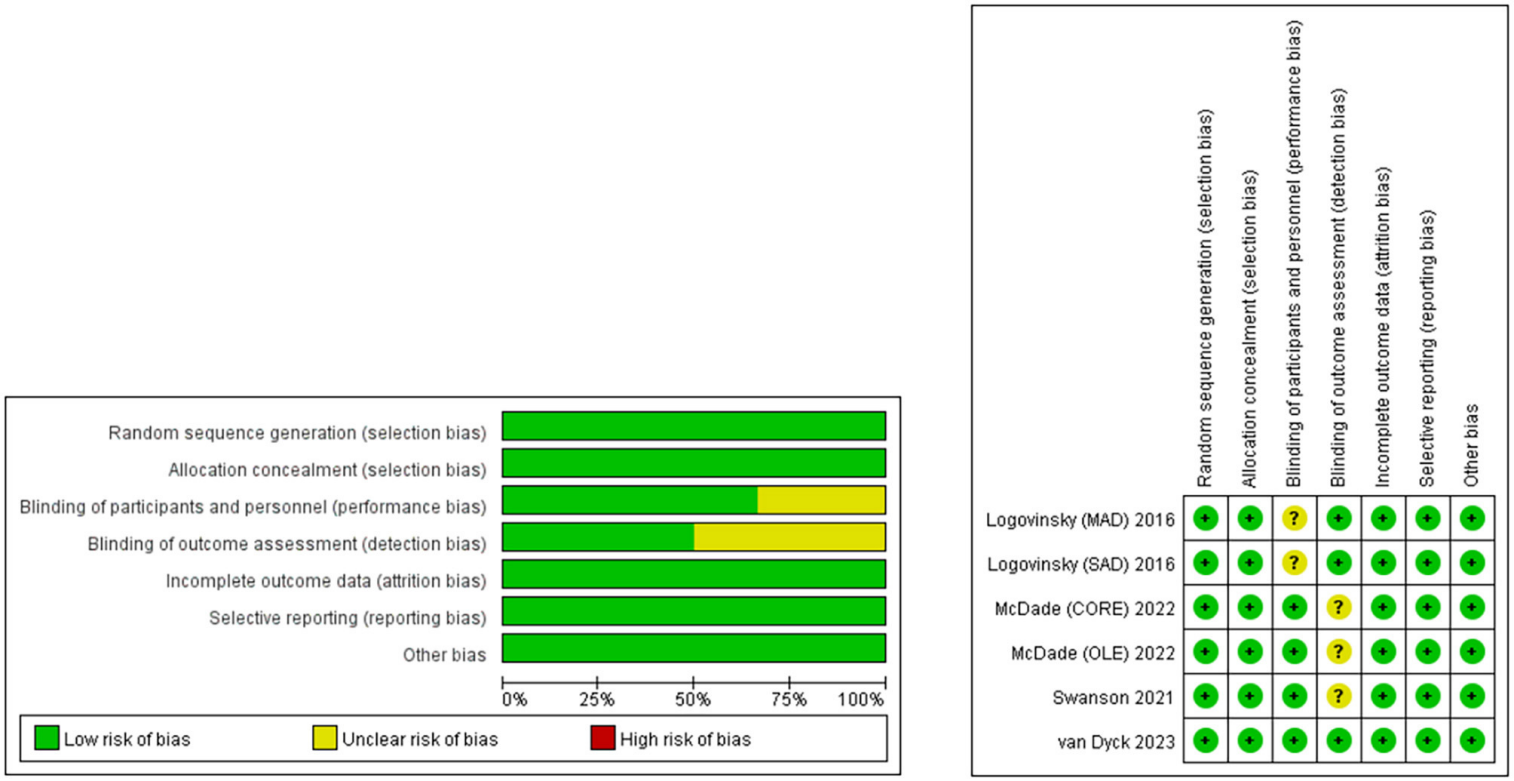
Risk of bias summary generated by RevMan software.

According to the Cochrane Handbook for Systematic Reviews of Interventions 5.1.0, the quality evaluation of RCT is based on the following aspects: random sequence generation, blind method of participants and personnel, blind method of result evaluation, incomplete result data, selective reports, and other biased sources (Cumpston et al., 2019). There are three bias levels in each research area, including low risk, high risk, and unclear. Studies with more “low-risk” bias assignations were regarded as superior. In total, two researchers independently evaluated the quality and level of qualified research evidence and solved the differences through discussion. Figure 1 shows the details of the quality assessment of all eligible studies.

### 2.5. Statistical analysis

Data synthesis was conducted in Review Manager (RevMan) software (version 5.3 for Windows) (Cochrane Collaboration, Oxford, UK). The WMD and OR were applied for the comparison of continuous and dichotomous variables, respectively, with a confidence interval of 95% in a meta-analysis model. The heterogeneity in studies was measured through the chi-square test, and the I-square test was used to quantify its extent. A chi-square *p* < 0.05 or *I* ^2^> 50% was considered a significant heterogeneity (Higgins and Thompson, 2002). In addition, we also conducted a one-way sensitivity analysis to evaluate the impact of the included studies on the comprehensive results with significant heterogeneity. The analysis is carried out under the stochastic effects model; otherwise, a fixed effect model was used. The funnel chart is created through Review Manager version 5.3, and the publication bias was evaluated intuitively. A *p* < 0.05 was considered a statistically significant publication bias (Lin, 2020).

## 3. Results

### 3.1. Study selection and characteristics

**Figure 2:**
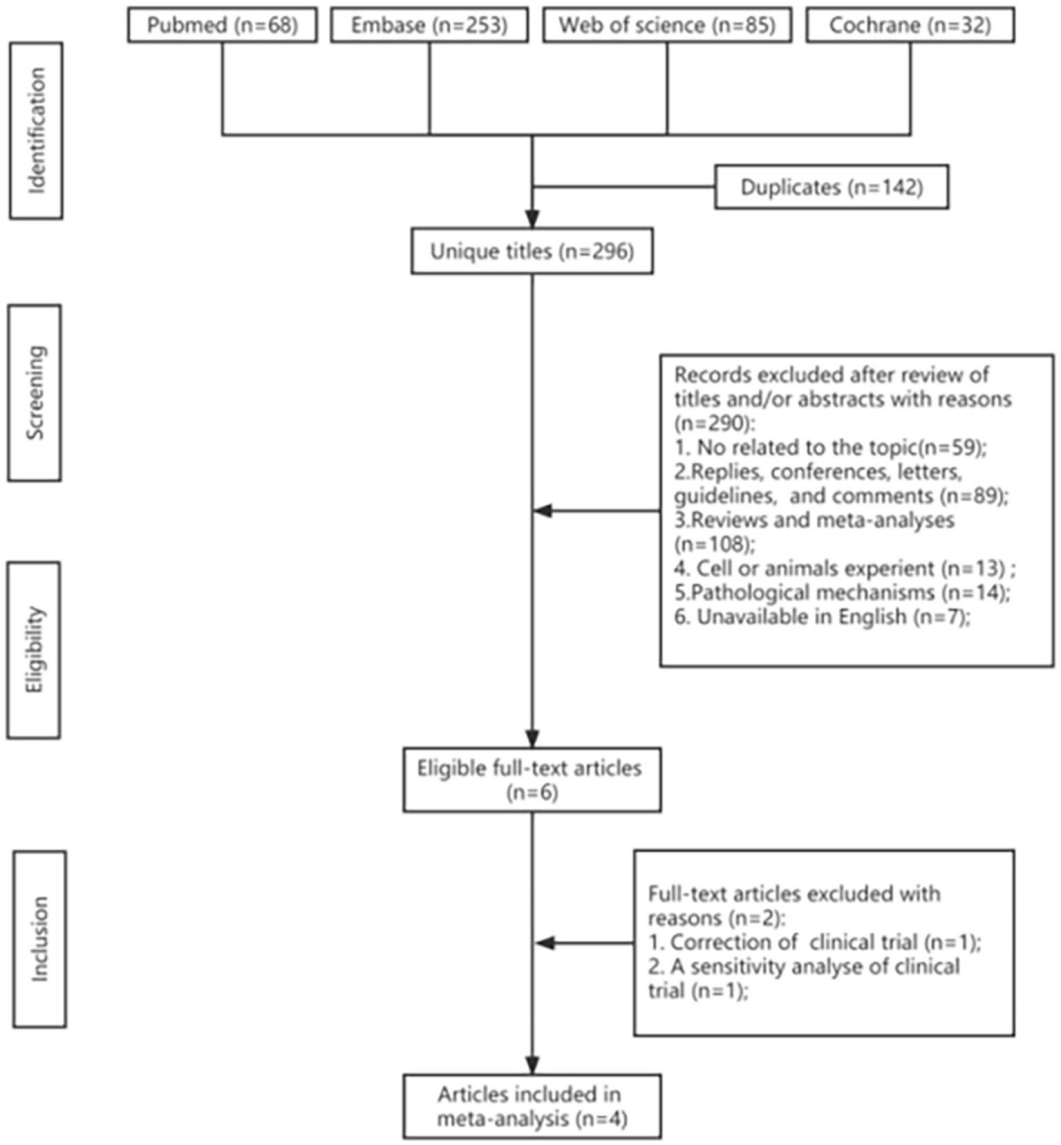
Flowchart of the systematic search and selection process.

A total of 406 potentially relevant articles were identified in the original systematic literature search. After removing duplicate studies, 282 titles and abstracts were reviewed. From 406 retrieved studies, of which four could be meta-analyzed, the flowchart of included studies is reported in Figure 2. Finally, four full-text articles involving 3,108 individuals with AD (1,695 in the lecanemab groups and 1,413 in the placebo groups) were included in the pooled analysis. All of these articles were RCTs (Lynch et al., 2019; Swanson et al., 2021; Mcdade et al., 2022; Van Dyck et al., 2023). Table 1 shows the detailed characteristics. In addition, the risk of bias in included studies was low according to the Cochrane risk of the bias assessment tool.

**Table 1:** Baseline features include research and methodology evaluation.

**References**	**Registration**	**Funding**	**Study design**	**Phase**	**Study period**	**Country**	**Severity of AD**	**Intervention**	**Follow-up period**
Logovinsky et al. (2016)	NCT01230853	No	Multicenter, double-blind randomized placebo-controlled study	1	2010-2012	USA	Mild to Moderate AD	SAD:Lecanemab and placebo: 0.1, 0.3, 1, 3, 10, and 15 mg/kg; MAD: Lecanemab and placebo: 0.3, 1, and 3 mg/kg (monthly) and 10 mg/kg (biweekly);	180 days
Swanson et al. (2021)	NCT01767311	Eisai Inc.	Multicenter, double-blind randomized placebo-controlled Bayesian design	2	2012-2017	North America, Europe, and Asia–Pacific	Mild AD	Lecanemab: 2.5, 5, and 10 mg/kg (biweekly) and 5 and 10 mg/kg (monthly); Placebo (biweeekly)	18 months
Mcdade et al. (2022)	NCT01767311	Eisai Inc.	Multicenter, double-blind, placebo-controlled, parallel-group study	2	2013–2022	North America, Europe, and Asia–Pacific	Mild AD	CORE: Lecanemab:2.5, 5, and 10 mg/kg (biweekly) and 5 and 10 mg/kg (monthly); Placebo (biweeekly) .OLE: 10 mg/kg biweekly	CORE: 3months;OLE:up to 24 months
Van Dyck et al. (2023)	NCT03887455	Eisai (regulatory sponsor), and Biogen.	Multicenter, double-blind, placebo-controlled, parallel-group trial	3	2019-2022	North America, Europe, and Asia–Pacific	Mild AD	Lecanemab and placebo: 10 mg/kg biweekly	18 months

SAD, single ascending dose; MAD, multiple ascending dose; CORE, study 201 blinded period; OLE, open-label extension.

### 3.2. Demographic variables

Demographic variables were analyzed according to the literature, and there were no significant differences between the two groups in terms of age (WMD: 0.5; 95% CI: −0.04, 1.04; *p* = 0.07), gender (female/total, OR: 0.87; 95% CI: 0.58, 1.33; *p* = 0.53), CDR 1.0 (OR: 0.91; 95% CI: 0.76, 1.11; *p* = 0.36), CDR 0.5 (OR: 1.08; 95% CI: 0.89, 1.31; *p* = 0.44), clinical subgroup (mild cognitive impairment, OR: 0.94; 95% CI: 0.81, 1.09; *p* = 0.42), clinical subgroup (mild dementia, OR: 1.07; 95% CI: 0.90, 1.28; *p* = 0.42), current use of medication for AD (OR: 0.97; 95% CI: 0.82, 1.13; *p* = 0.66), CDR-SB (OR: 0; 95% CI: −0.10, 0.10; *p* = 0.97), amyloid burden on PET (OR:−22.67; 95% CI:−55.63, 1.029; *p* = 0.18), ADAS-cog14 score (OR: −0.11; 95% CI: −0.66, 0.44; *p* = 0.70), and ADCOMS (OR: 0; 95% CI: −0.01, 0.01; *p* = 0.90). However, the two groups were significantly different in baseline characteristics in terms of ApoE4 status (non-carrier/total, OR: 2.87; 95% CI: 1.10, 7.53; *p* = 0.03) and MMSE (WMD: 0.2; 95% CI: −0.36, −0.04; *p* = 0.02) (Table 2).

**Table 2:** Demographic and clinical features included in the studies.

**Outcomes**	**Studies**	**No. of patients**	**WMD or OR**	**95% CI**	***p*-value**	**Heterogeneity**			
		**Lecanemab/ Placebo**				**Chi** ^2^	**df**	***p*** **-value**	**I** ^2^ **(%)**
Age (years)	6	1,695/1,413	0.5	[−0.04, 1.04]	0.07	9.55	5	0.09	48
Gender (female)	6	1,695/1,413	0.87	[0.58, 1.33]	0.53	21.48	5	0.0007	77
MMSE	4	1,635/1,393	−0.2	[−0.36, −0.04]	0.02^a^	2.88	3	0.41	0
CDR 1.0	4	1,635/1,393	0.91	[0.76, 1.11]	0.36	1.97	3	0.58	0
CDR 0.5	4	1,635/1,393	1.08	[0.89, 1.31]	0.44	1.04	3	0.79	0
Clinical subgroup (Mild cognitive impairment)	4	1,635/1,393	0.94	[0.81, 1.09]	0.42	0.87	3	0.83	0
Clinical subgroup (Mild dementia)	3	1,048/1,155	1.07	[0.90, 1.28]	0.42	0.83	2	0.66	0
ApoE4 status (Noncarrier)	4	1,635/1,393	2.87	[1.10, 7.53]	0.03 ^a^	71.99	3	< 0.00001	96
Current use of medication for AD	2	1,446/1,113	0.97	[0.82, 1.13]	0.66	0.20	1	0.66	0
CDR-SB	4	1,635/1,393	0	[−0.10, 0.10]	0.97	1.98	3	0.58	0
Amyloid burden on PET	3	925/1,001	−22.67	[-55.63, 10.29]	0.18	46.14	2	< 0.00001	96
ADAS- cog14 score	4	1,634/1,392	−0.11	[−0.66, 0.44]	0.70	0.84	3	0.84	0
ADCOMS	4	1,635/1,393	0	[−0.01, 0.01]	0.90	0.67	3	0.88	0

a Statically significant.

MMSE, Mini–Mental State Examination; CDR, Global Clinical Dementia Rating; CDR-SB, CDR Sum of Boxes; ADAS-cog14, Alzheimer's Disease Assessment Scale; ADCOMS, Alzheimer's Disease Composite Score; WMD, weighted mean difference; OR, odds ratio; CI, confidence interval.

### 3.3. Effects of interventions

#### 3.3.1. Change of clinical dementia rating-sum-of-boxes (CDR-SB)

**Figure 3:**
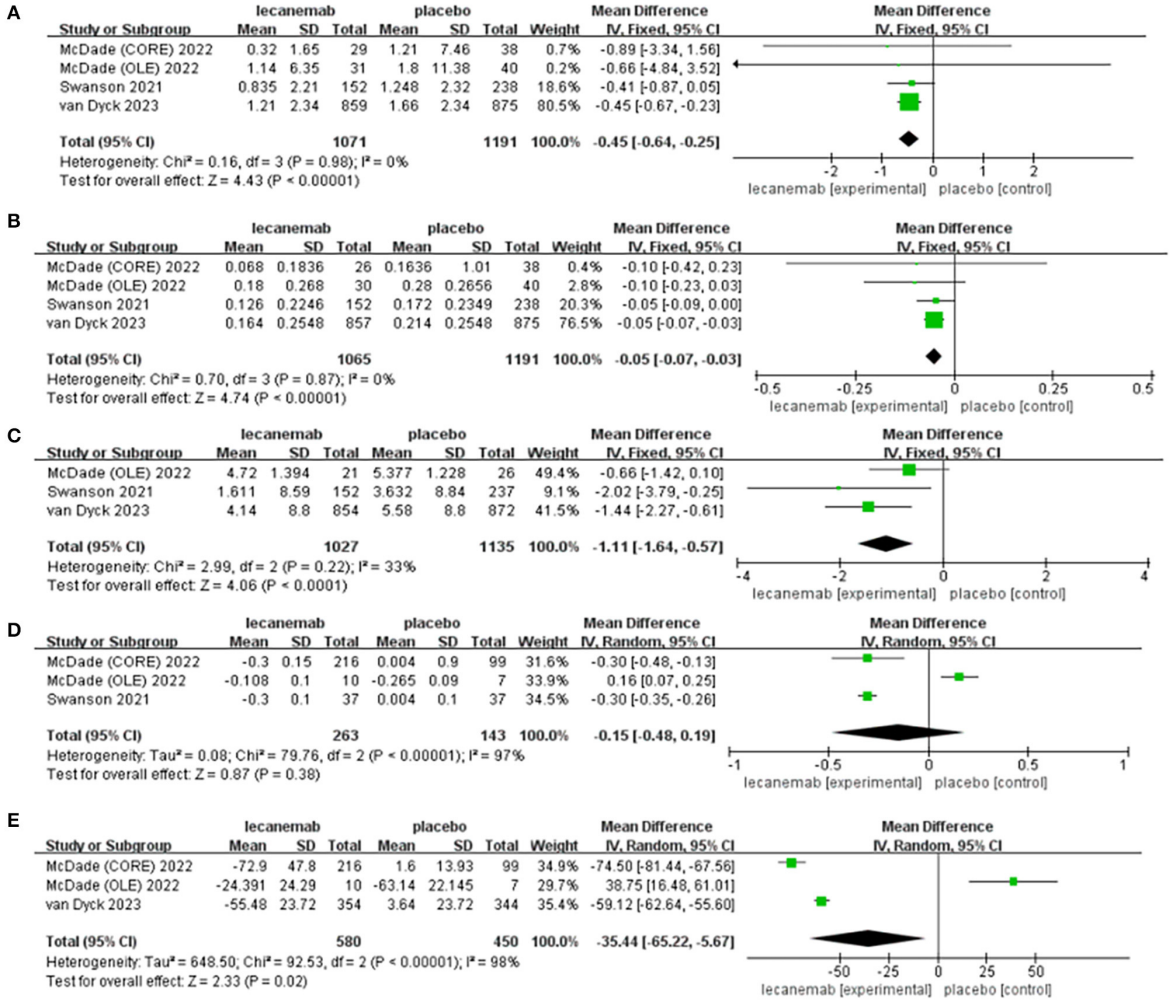
Forest plot of mean difference (MD) in **(A)** CDR-SB, **(B)** ADCOMS, **(C)** ADAS-cog14 score, **(D)** Amyloid PET SUVr, and **(E)** amyloid burden on PET.

**Figure 4:**
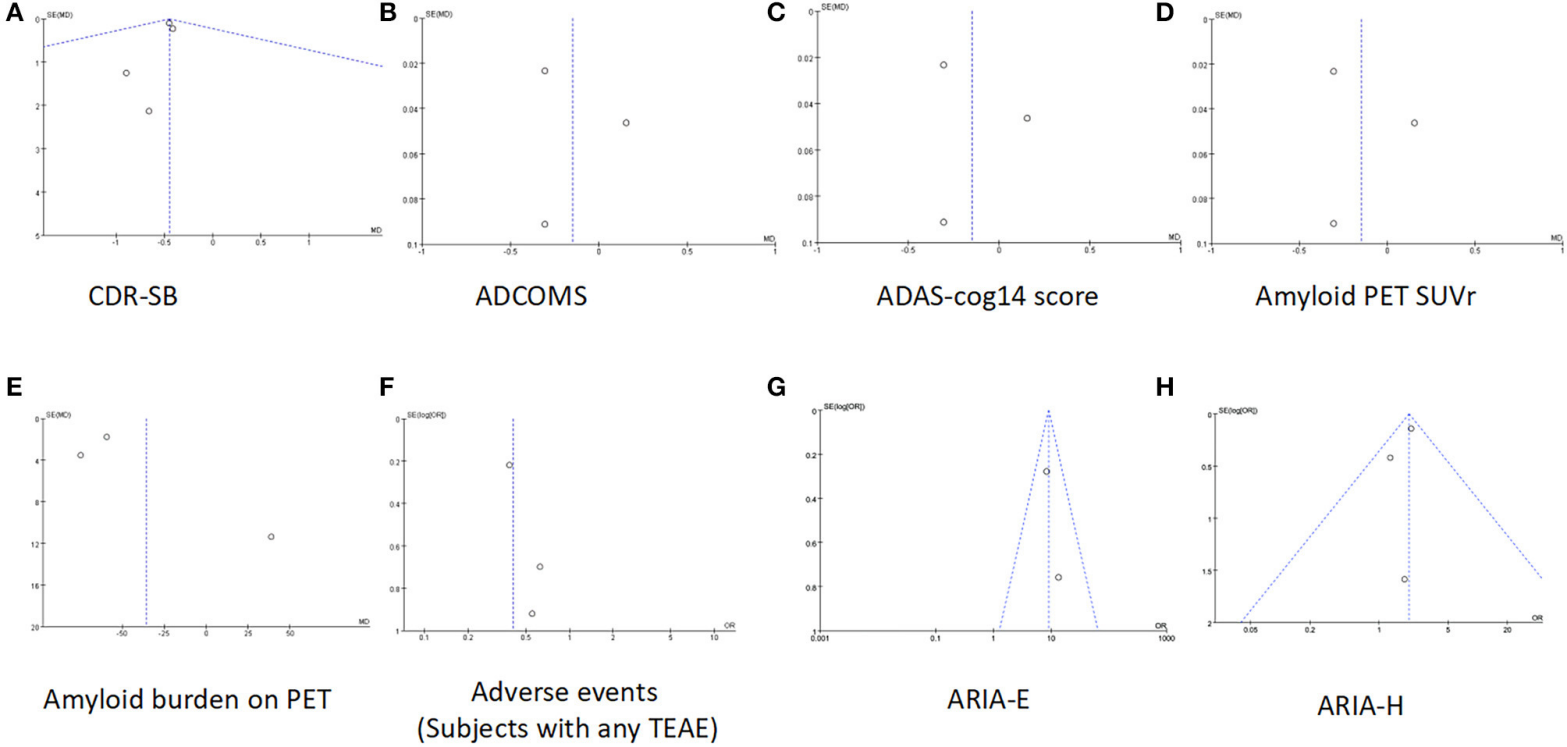
Funnel plots of **(A)** CDR-SB, **(B)** ADCOMS, **(C)** ADAS-cog14 score, **(D)** Amyloid PET SUVr, **(E)** amyloid burden on PET, **(F)** adverse events (subjects with any TEAE), **(G)** ARIA-E, and **(H)** ARIA-H.

Data on CDR-SB were synthesized from four studies including 2,262 patients (1,071in lecanemab vs. 1,191 in placebo) (Mcdade et al., 2022; Van Dyck et al., 2023). Among the available data, the cognitive effects of all drugs were displayed by meta-analysis, the pooled WMD between lecanemab and placebo in CDR-SB was significant (WMD: −0.45; 95% CI: −0.64, −0.25; *p* < 0.00001), and no significant heterogeneity was observed (I^2^ = 0%, *p* = 0.98) (Figure 3A). Funnel plots revealed a slight publication bias (Figure 4A).

#### 3.3.2. Change of ADCOMS

The overall effect estimate showed that lecanemab significantly reduced the score of ADCOMS at the treatment endpoint compared to the placebo (WMD: −0.05; 95% CI: −0.07, −0.03; *p* < 0.00001) which suggests that lecanemab could reduce the impairment of cognition (Figure 3B) (Mcdade et al., 2022; Van Dyck et al., 2023). The heterogeneity among most pooled studies was low (*p* = 0.87, I ^2^ = 0%), and the funnel plot seemed symmetric and did not show obvious publication bias (Figure 4B).

#### 3.3.3 ADAS-cog14 score

We pooled the data of ADAS-cog14 score from three articles [18, 26, 27], and significant benefit was found with lecanemab treatment (WMD: −1.11; 95% CI: −1.64, −0.57; *p* < 0.0001), and no significant heterogeneity was observed (*p* = 0.22, I^2^ = 33%) (Figure 3C). A visual assessment of the funnel plot did not indicate the presence of publication bias (Figure 4C).

#### 3.3.4. Change of amyloid PET SUVr

Analysis of amyloid PET SUVr was conducted in two studies with 406 patients (263 lecanemab vs. 143 placebo) (Swanson et al., 2021; Mcdade et al., 2022; Van Dyck et al., 2023). Pooled analysis showed no significant difference of SUVr between the lecanemab group and the placebo group (WMD: −0.15; 95% CI: −0.48, 0.19; *p* = 0.38) with a statistically significant heterogeneity (I ^2^ = 97%, *p* < 0.00001) (Figure 3D). The funnel plot was relatively symmetric and did not suggest the presence of publication bias (Figure 4D).

#### 3.3.5. Change of amyloid burden on PET—centiloids

In total, two studies involving 1,030 patients (580 patients with lecanemab and 450 patients with placebo) were included in the analysis (Mcdade et al., 2022; Van Dyck et al., 2023). Summary analysis showed that the load of PET amyloid protein in the lecanemab group was significantly increased (WMD:−35.44; 95% CI: −65.22, −5.67; *P* =0.02), which has obvious heterogeneity **(**I ^2^ = 98%, *p* < 0.00001) (Figure 3E). A visual evaluation of the funnel diagram shows that there is a slight publishing deviation (Figure 4E).

#### 3.3.6. Adverse events (subjects with any TEAE)

**Figure 5:**
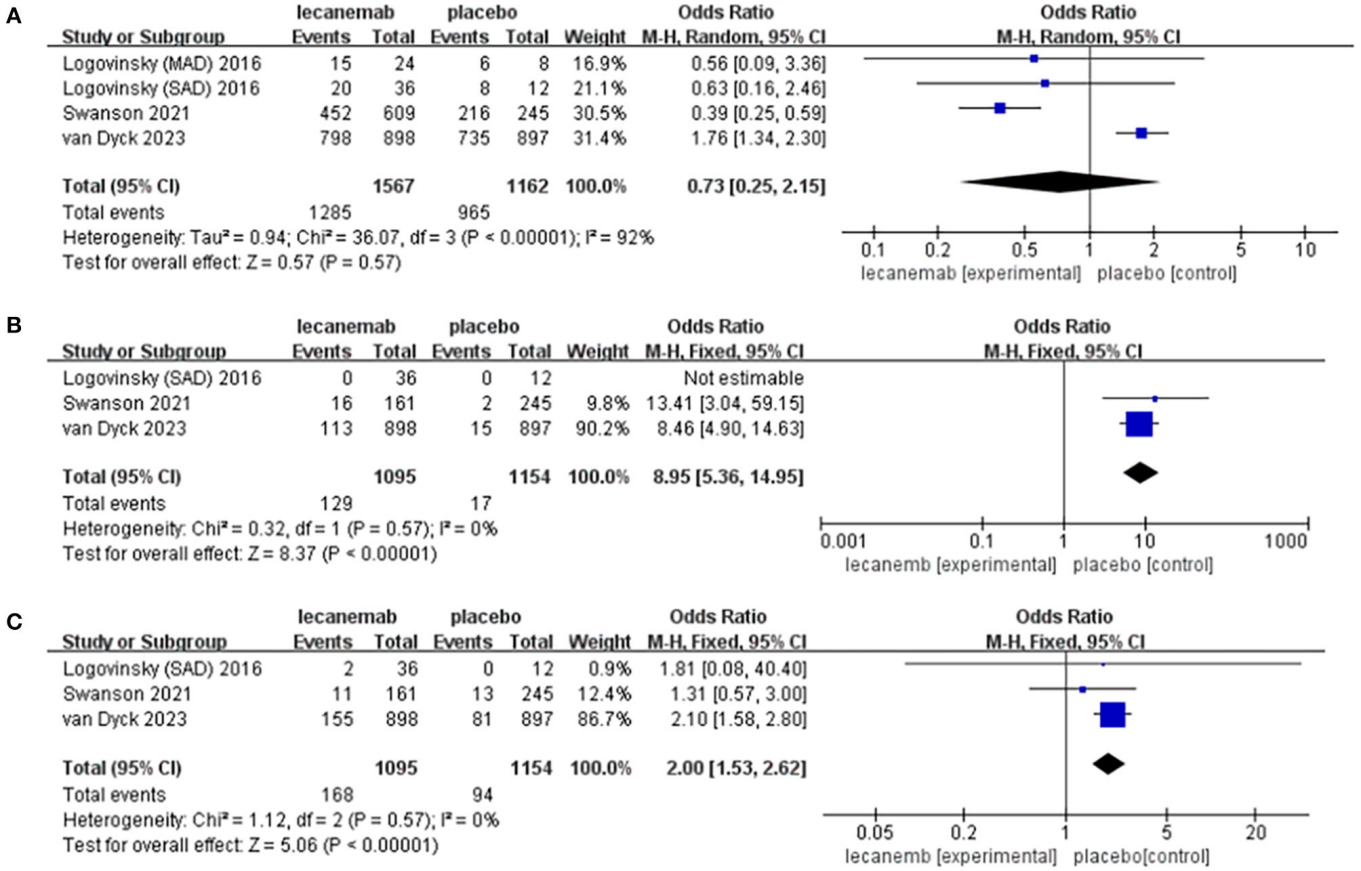
Forest plot of safety endpoints: **(A)** adverse events (subjects with any TEAE), **(B)** ARIA-E, and **(C)** ARIA-H.

In total, four articles reported adverse events during the trial, including 2,729 patients (1,567 lecanemab vs. 1,162 placebo) (Logovinsky et al., 2016; Swanson et al., 2021; Van Dyck et al., 2023). There was no significant difference between the two groups (OR: 0.73; 95% CI: 0.25, 2.15; *p* = 0.57), but statistically significant heterogeneity was observed (I 2 = 92%, *p* < 0.00001) (Figure 5A). The funnel diagram does not show symmetric distribution, which implies publication bias (Figure 4F).

#### 3.3.7. ARIA-E

In total, three studies with 2,249 patients (1,095 lecanemab vs. 1,154 placebo) were included in the analysis of the events with ARIA-E (Logovinsky et al., 2016; Van Dyck et al., 2023). The summary analysis showed that the incidence of ARIA-E was significantly higher in the lecanemab group (OR: 8.95; 95% CI: 5.36, 14.95; *p* < 0.00001) (Figure 5B). No significant heterogeneity (I ^2^ = 0%, *p* = 0.57) or visual evidence of publication bias was observed (Figure 4G).

#### 3.3.8. ARIA-H

In total, three articles were included in the analysis for the events of ARIA-H, involving 2,249 patients (1,095 in lecanemab vs. 1,154 in placebo) (Logovinsky et al., 2016; Van Dyck et al., 2023). Evidence synthesis showed that the lecanemab group had a higher frequency in ARIA-H (OR: 2.00; 95% CI: 1.53, 2.62; *p* < 0.00001) with no significant heterogeneity (I ^2^ = 0%, *p* =0.57) being detected (Figure 5C). The funnel plot (Figure 4H) did not detect publication bias.

### 3.4. Sensitivity analysis

**Figure 6:**
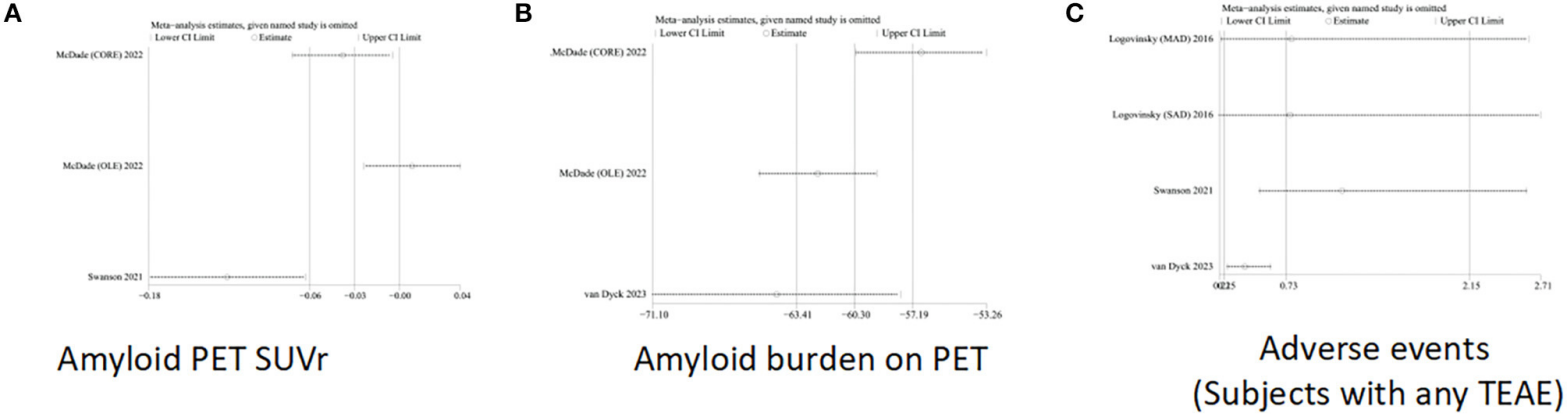
Sensitivity analysis of **(A)** Amyloid PET SUVr, **(B)** amyloid burden on PET, and **(C)** adverse events (subjects with any TEAE).

One-way sensitivity analyses were conducted to compare the burden of amyloid protein PET SUVr and amyloid protein on PET and adverse events (subjects had any TEAE) so as to evaluate the impact of a single study on joint WMD through removing single studies one by one. Sensitivity analyses showed that the new combined WMD remained unchanged after excluding any separate study on amyloid loading of PET (Figure 6B). However, when we excluded the data of open-label extension reported by McDade et al. in 2022, the heterogeneity of amyloid protein PET SUVr disappeared (I ^2^ = 0%, *p* = 1.0) (Figure 6A), indicating that most of the heterogeneity was explained by this study. In addition, when the study of Swanson et al. was excluded, the effect size for adverse events (subjects with any TEAE) decreased (I ^2^ = 0%, *p* = 0.76) (Figure 6C) in the sensitivity analysis.

## 4. Discussion

The meta-analysis included data from all reported RCTs and compared the efficacy of lecanemab and placebo in the treatment of cognitive decline in sporadic AD. This study, the first of its kind that we know, is based on the safety and effectiveness of this monoclonal antibody in patients with mild-to-moderate AD. A previous meta-analysis and systematic review that studied the effect of amyloid on cognitive decline and the risk-benefit profile of different monoclonal antibodies only included phase I and IIb clinical research studies. Compared to this research, we added two latest studies and performed a meta-analysis and sensitivity analyses on lecanemab to make the results more credible (Lacorte et al., 2022). The reliable data of all included studies (2,262 participants) show that the cognitive results of early AD (such as CDR-SB, ADCOMS, and ADAS-Cog) have improved statistically. The statistically significant cognitive benefits of monoclonal antibodies revealed in this meta-analysis were particularly noteworthy considering that the majority of original studies did not reach significance on ADAS-Cog. Sensitivity analyses of the amyloid burden of PET are usually consistent with a preliminary analysis although the source of heterogeneity was still unclear. In addition, sensitivity analyses were carried out to evaluate the stability of amyloid PET SUVr, and the source of heterogeneity mainly came from the open-label extension trial. Consistent with other results, a decrease in the value of amyloid PET SUVr was observed in the lecanemab group. Swanson et al. found that lecanemab treatment produced consistent dose-dependent reductions in clinical decline and brain amyloid burden in patients with early AD. In this study, the dose of 10 mg/kg lecanemab injected intravenously every 2 weeks is considered to be the best dose to test the Aβ clearance rate, clinical efficacy, and safety in the phase 3 clinical trial.

APOE genotype is a risk factor for AD, and APOE-ε 4 allele is considered to be the strongest genetic modifier of late-onset Alzheimer's disease (Corder et al., 1993; Neu et al., 2017). Lecanemab has been proven to be a high-risk factor for ARIA, thus, researchers stopped giving high-dose lecanemab to homozygous and heterozygous APOE 4 carriers, which led to a complicated explanation of results in 2022 (Van Dyck et al., 2023). According to these studies, the positive effects of lecanemab groups are overestimated because individuals with higher MMSE scores actually progress more slowly. The APOE-e 4 individuals, particularly the homozygotes, have limited therapeutic effectiveness with the drug treatment and have substantially more ARIA. Furthermore, recent studies have shown that the degree of brain atrophy increases when amyloid is removed, which may be related to the decrease in the PET SUVr value of amyloid (Klunk et al., 2004; Pereira et al., 2021). The amyloid cascade hypothesis postulates that the amyloid precursor protein is broken down to form Aβ, which results in an abnormal accumulation of amyloid-beta plaques in various regions of the brain. This hypothesis is very important because the formation of the Aβ monomer could lead to the deposition of extracellular fibers, leading to neuronal death and the formation of senile plaques (Hardy and Higgins, 1992; Multhaup et al., 2015; Fulop et al., 2018). In fact, there is a lack of a correlation between the level of PET amyloid in the brain or even the estimated value of amyloid in the brain after death and cognitive function, indicating that the amyloid-β itself is a pathological sign of AD, not the cause of the AD process, nor the cause of cognitive impairment of AD (Morris et al., 2009; Adlard et al., 2014). In addition, the progress of drug development is not satisfactory because the research mainly aimed at binding Aβ monomer (solanezumab and crenezumab) or the mixture of monomer and plaques (bapineuzumab) failed to show clinical efficacy (Cummings et al., 2018; Honig et al., 2018; Long and Holtzman, 2019).

In clinical research, safety and tolerance are equally important as the effectiveness of interventions. Several articles proposed that amyloid-β assumes major responsibility for anti-pathogenic functionalities as a barrier protein with a unique sealant that prevents immune-mediated tissue damage of the brain. Therefore, the danger of insufficient amyloid-β can lead to a series of complications such as downstream hemorrhage and edema that should be brought to attention (Atwood and Perry, 2023). In the current meta-analysis, compared with the placebo group, lecanemab significantly increases the risk of ARIA-E and ARIA-H. Even though the incidence of ARIA, including symptomatic ARIA, was numerically lower than in similar clinical trials, differences between drugs used and trial design are not allowed to be directly compared. In addition, significant heterogeneity of adverse events (subjects with any TEAE) mainly comes from study 201, and we inferred that this was due to Swanson et al. in the early stage of Alzheimer's disease, three doses of lecanemab from two schemes were compared with placebo, but we counted all the adverse reactions of the results. Compared to other monoclonal antibodies from the published meta-analysis, lecanemab shows the prospect of a strong signal of consistency in efficacy and direction between reducing Aβ amyloid and slowing cognitive decline. It also shows the potential for fewer ARIA because of the Aβ species targeted (Abushouk et al., 2017; Lu et al., 2020). Among these side effects, some gastrointestinal and nervous system side effects have been observed, such as nausea, vomiting, diarrhea, anorexia, dizziness, depression, and headache. Since some studies have not reported these events in detail, we did not compare the incidence of AE separately.

Our study reports the latest and largest evidence-based analysis for the first time and evaluates the early Alzheimer's disease subjects who received lecanemab treatment. All randomized controlled trials in our research are considered to be of high quality. However, we have to admit some limitations at present. First, the generalization of our results can be limited by the small number of available trials and the number of patients provided is relatively small; three RCTs were funded by Eisai Inc., which may report selective data for positive clinical effects and result in publication bias. Second, we mainly compare the changes in cognitive function in clinical results. Most of the measurement scales used for testing the benefits of lecanemab were outdated, including MMSE, which has been shown to be an unreliable test given its non-linear relationship with time course, especially before moderate dementia (Fotuhi et al., 2009; Thomas et al., 2016). In addition, the true meaning of the change observed on CDR-SB is doubtful because it has never been correctly analyzed in relation to the time change and the great possibility of random effect (Morris, 1993; O'Bryant et al., 2008). Unfortunately, lecanemab or any other anti-amyloid drugs have not been evaluated satisfactorily. Each research variable is different in key biomarkers, thus, it is impossible to compare. Finally, meta-analysis data come only from published scientific literature, and some negative results and non-statistical data are difficult to publish. Therefore, there is publication bias. We believe that this is unlikely to affect our results because all studies analyzed their data by intention therapy. In general, these results show that lecanemab could slow the decline of cognitive ability of patients with early AD but the curative effect on the symptoms of functional, behavioral, and systemic change of patients with severe AD is questionable. Indeed, some outcomes are difficult to evaluate today due to immature tools for manipulating non-cognitive outcomes (Cotta Ramusino et al., 2021). Our results might provide a possible perspective for anti-dementia drug trials, such as the increase of placebo effects and heterogeneity of neuropsychiatric symptoms in AD over time. We only pay attention to monoclonal antibodies against Aβ, and we need to consider more directions for treating Alzheimer's disease in the future, including gene therapy, immunotherapy, peptide mimetics, metal creators, and probiotics. According to the meta-analysis of lecanemab in existing studies, lecanemab has clinical benefits in the treatment of AD, but whether it is really worth popularizing in clinical use remains to be further discussed.

The above analysis was conducted to evaluate the efficacy of lecanemab compared with a placebo. We performed all steps in strict accordance with the Cochrane handbook of systematic reviews and reported them according to the preferred reporting items for systematic reviews and meta-analysis (PRISMA) statement guidelines.

## 5. Conclusion

Our analysis is the first attempt to combine available direct or indirect evidence to evaluate the effectiveness and safety of this latest drug lecanemab in the treatment of AD. The results showed that lecanemab showed significant positive effects on cognition, function, behavior, and overall change. However, the existing randomized controlled trials were only conducted in patients with mild or early AD. Further studies are needed to be implemented targeting the global population with a large sample size and an extended period to evaluate whether lecanemab could be utilized as a potential disease-modifying treatment for advanced AD patients.

## Data availability statement

The original contributions presented in the study are included in the article/Supplementary material, further inquiries can be directed to the corresponding author.

## Author contributions

YQ: subject design and writing—original draft. YC and QZ: investigation. YM: conceptualization, supervision, and funding acquisition—reviewing and editing. All authors contributed to the article and approved the submitted version.

## References

[B1] (2022). 2022 Alzheimer's disease facts and figures. Alzheimers. Dement. 18, 700–789. 10.1002/alz.12638 35289055

[B2] Abushouk A. I. Elmaraezy A. Aglan A. Salama R. Fouda S. Fouda R. . (2017). Bapineuzumab for mild to moderate Alzheimer's disease: a meta-analysis of randomized controlled trials. BMC Neurol. 17, 66. 10.1186/s12883-017-0850-1 28376794 PMC5381133

[B3] Adlard P. A. Tran B. A. Finkelstein D. I. Desmond P. M. Johnston L. A. Bush A. I. . (2014). A review of β-amyloid neuroimaging in Alzheimer's disease. Front. Neurosci. 8, 327. 10.3389/fnins.2014.00327 25400539 PMC4215612

[B4] Atwood C. S. Perry G. (2023). Russian roulette with alzheimer's disease patients: do the cognitive benefits of lecanemab outweigh the risk of edema and stroke? J. Alzheimers Dis. 92, 799–801. 10.3233/JAD-230040 36847013

[B5] Ayaz M. Junaid M. Ullah F. Sadiq A. Khan M. A. Ahmad W. . (2015). Comparative chemical profiling, cholinesterase inhibitions and anti-radicals properties of essential oils from Polygonum hydropiper L: a preliminary anti- Alzheimer's study. Lipids Health Dis. 14, 141. 10.1186/s12944-015-0145-8 26530857 PMC4632677

[B6] Barthel H. (2023). Amyloid imaging-based food and drug administration approval of lecanemab to treat alzheimer disease-what lasts long finally becomes good? J. Nucl. Med. 64, 503–504. 10.2967/jnumed.123.265667 36958857

[B7] Corder E. H. Saunders A. M. Strittmatter W. J. Schmechel D. E. Gaskell P. C. Small G. W. . (1993). Gene dose of apolipoprotein E type 4 allele and the risk of Alzheimer's disease in late onset families. Science. 261, 921–923. 10.1126/science.8346443 8346443

[B8] Cotta Ramusino M. Perini G. Altomare D. Barbarino P. Weidner W. Salvini Porro G. . (2021). Outcomes of clinical utility in amyloid-PET studies: state of art and future perspectives. J. Alzheimers Dis. 48, 2157–2168. 10.1007/s00259-020-05187-x 33594474 PMC8175294

[B9] Cummings J. L. Cohen S. van Dyck C. H. Brody M. Curtis C. Cho W. . (2018). ABBY: A phase 2 randomized trial of crenezumab in mild to moderate Alzheimer disease. Neurology. 90, e1889. 10.1212/WNL.0000000000005550 29695589 PMC5962917

[B10] Cummings J. L. Tong G. Ballard C. (2019). Treatment combinations for Alzheimer's disease: current and future pharmacotherapy options. J. Alzheimers. Dis. 67, 779–794. 10.3233/JAD-180766 30689575 PMC6398562

[B11] Cumpston M. LI T. Page M. J. Chandler J. Welch V. A. Higgins J. P. . (2019). Updated guidance for trusted systematic reviews: a new edition of the Cochrane Handbook for Systematic Reviews of Interventions. Cochrane Database Syst Rev. 10, 000142. 10.1002/14651858.ED000142 31643080 PMC10284251

[B12] Englund H. Sehlin D. Johansson A. S. Nilsson L. N. Gellerfors P. Paulie S. . (2007). Sensitive ELISA detection of amyloid-beta protofibrils in biological samples. J. Neurochem. 103, 334–345. 17623042 10.1111/j.1471-4159.2007.04759.x

[B13] Fotuhi M. Hachinski V. Whitehouse P. J. (2009). Changing perspectives regarding late-life dementia. Nat. Rev. Neurol. 5, 649–658. 10.1038/nrneurol.2009.175 19918254

[B14] Fulop T. Witkowski J. M. Bourgade K. Khalil A. Zerif E. Larbi A. . (2018). Can an infection hypothesis explain the beta amyloid hypothesis of Alzheimer's disease? Front. Aging Neurosci. 10, 224. 10.3389/fnagi.2018.00224 30087609 PMC6066504

[B15] Gustavsson T. Syvanen S. O'callaghan P. Sehlin D. (2020). SPECT imaging of distribution and retention of a brain-penetrating bispecific amyloid-beta antibody in a mouse model of Alzheimer's disease. Transl. Neurodegener. 9, 37. 10.1186/s40035-020-00214-1 32951598 PMC7504681

[B16] Hardy J. A. Higgins G. A. (1992). Alzheimer's disease: the amyloid cascade hypothesis. Science. 256, 184–185. 10.1126/science.1566067 1566067

[B17] Higgins J. P. Thompson S. G. (2002). Quantifying heterogeneity in a meta-analysis. Stat. Med. 21, 1539–1558. 10.1002/sim.1186 12111919

[B18] Honig L. S. Vellas B. Woodward M. Boada M. Bullock R. Borrie M. . (2018). Trial of solanezumab for mild dementia due to Alzheimer's Disease. N. Engl. J. Med. 378, 321–330. 10.1056/NEJMoa1705971 29365294

[B19] Hozo S. P. Djulbegovic B. Hozo I. (2005). Estimating the mean and variance from the median, range, and the size of a sample. BMC Med. Res. Methodol. 5, 13. 10.1186/1471-2288-5-13 15840177 PMC1097734

[B20] Ising C. Venegas C. Zhang S. Scheiblich H. Schmidt S. V. Vieira-Saecker A. . (2019). NLRP3 inflammasome activation drives tau pathology. Nature. 575, 669–673. 10.1038/s41586-019-1769-z 31748742 PMC7324015

[B21] Klunk W. E. Engler H. Nordberg A. Wang Y. Blomqvist G. Holt D. P. . (2004). Imaging brain amyloid in Alzheimer's disease with Pittsburgh Compound-B. Ann. Neurol. 55, 306–319. 10.1002/ana.20009 14991808

[B22] Lacorte E. Ancidoni A. Zaccaria V. Remoli G. Tariciotti L. Bellomo G. . (2022). Safety and efficacy of monoclonal antibodies for Alzheimer's disease: a systematic review and meta-analysis of published and unpublished clinical trials. J. Alzheimer's Dis. 87, 101–129. 10.3233/JAD-220046 35275549 PMC9198746

[B23] Lin L. (2020). Hybrid test for publication bias in meta-analysis. Stat. Methods Med. Res. 29, 2881–2899. 10.1177/0962280220910172 32290810 PMC7434640

[B24] Logovinsky V. Satlin A. Lai R. Swanson C. Kaplow J. Osswald G. . (2016). Safety and tolerability of BAN2401 - A clinical study in Alzheimer's disease with a protofibril selective Aβ antibody. Alzheimer's Res. Ther. 8. 10.1186/s13195-016-0181-2 27048170 PMC4822297

[B25] Long J. M. Holtzman D. M. (2019). Alzheimer disease: an update on pathobiology and treatment strategies. Cell. 179, 312–339. 10.1016/j.cell.2019.09.001 31564456 PMC6778042

[B26] Lord A. Gumucio A. Englund H. Sehlin D. Sundquist V. S. Söderberg L. . (2009). An amyloid-beta protofibril-selective antibody prevents amyloid formation in a mouse model of Alzheimer's disease. Neurobiol. Dis. 36, 425–434. 10.1016/j.nbd.2009.08.007 19703562

[B27] Lu L. Zheng X. Wang S. Tang C. Zhang Y. Yao G. . (2020). Anti-Aβ agents for mild to moderate Alzheimer's disease: systematic review and meta-analysis. J. Neurol. Neurosurg. Psychiatr. 91, 1316–1324. 10.1136/jnnp-2020-323497 33046560

[B28] Lynch S. Y. Irizarry M. Dhadda S. Zhang Y. Wang J. Bogoslovsky T. . (2019). BAN2401 in early Alzheimer's disease: a placebo-controlled, double-blind, parallel-group, 18-month study with an open-label extension phase to confirm safety and efficacy (clarity AD). J. Prevent. Alzheimer's Dis. 6, S145–S146.

[B29] Mcdade E. Cummings J. L. Dhadda S. Swanson C. J. Reyderman L. Kanekiyo M. . (2022). Lecanemab in patients with early Alzheimer's disease: detailed results on biomarker, cognitive, and clinical effects from the randomized and open-label extension of the phase 2 proof-of-concept study. Alzheimer's Res. Ther. 14, 191. 10.1186/s13195-022-01124-2 36544184 PMC9768996

[B30] Morris J. C. (1993). The clinical dementia rating (CDR): current version and scoring rules. Neurology. 43, 2412–2414. 10.1212/WNL.43.11.2412-a 8232972

[B31] Morris J. C. Roe C. M. Grant E. A. Head D. Storandt M. Goate A. M. . (2009). Pittsburgh compound B imaging and prediction of progression from cognitive normality to symptomatic Alzheimer disease. Arch. Neurol. 66, 1469–1475. 10.1001/archneurol.2009.269 20008650 PMC2798814

[B32] Multhaup G. Huber O. Buée L. Galas M. C. (2015). Amyloid precursor protein (APP) metabolites APP intracellular fragment (AICD), Aβ42, and tau in nuclear roles. J. Biol. Chem. 290, 23515–23522. 10.1074/jbc.R115.677211 26296890 PMC4583011

[B33] Neu S. C. Pa J. Kukull W. beekly D. Kuzma A. Gangadharan P. . (2017). Apolipoprotein E genotype and sex risk factors for alzheimer disease: a meta-analysis. JAMA Neurol. 74, 1178–1189. 10.1001/jamaneurol.2017.2188 28846757 PMC5759346

[B34] Nikitidou E. Söllvander S. Zyśk M. Söderberg L. Sehlin D. Lannfelt L. . (2017). The Aβ protofibril selective antibody mAb158 prevents accumulation of Aβ in astrocytes and rescues neurons from Aβ induced apoptosis. GLIA. 65, E170. 10.1186/s12974-018-1134-4 PMC5875007 29592816

[B35] O'Bryant S. E. Waring S. C. Cullum C. M. Hall J. Lacritz L. . (2008). Staging dementia using clinical dementia rating scale sum of boxes scores: a Texas alzheimer's research consortium study. Arch. Neurol. 65, 1091–1095. 10.1001/archneur.65.8.1091 18695059 PMC3409562

[B36] Page M. J. Mckenzie J. E. Bossuyt P. M. Boutron I. Hoffmann T. C. Mulrow C. D. . (2021). The PRISMA 2020 statement: an updated guideline for reporting systematic reviews. BMJ. 372, n,71. 10.31222/osf.io/v7gm2 33782057 PMC8005924

[B37] Panza F. Lozupone M. Logroscino G. Imbimbo B. P. (2019). A critical appraisal of amyloid-β-targeting therapies for Alzheimer disease. Nature Reviews Neurology 15, 73–88. 10.1038/s41582-018-0116-6 30610216

[B38] Pereira J. B. Janelidze S. Stomrud E. Palmqvist S. Van Westen D. Dage J. L. . (2021). Plasma markers predict changes in amyloid, tau, atrophy and cognition in non-demented subjects. Brain. 144, 2826–2836. 10.1093/brain/awab163 34077494 PMC8557344

[B39] Sehlin D. Englund H. Simu B. Karlsson M. Ingelsson M. Nikolajeff F. . (2012). Large aggregates are the major soluble Aβ species in AD brain fractionated with density gradient ultracentrifugation. PLoS ONE. 7, e,32014. 10.1371/journal.pone.0032014 22355408 PMC3280222

[B40] Sehlin D. Fang X. T. Cato L. Antoni G. Lannfelt L. . (2016). Antibody-based PET imaging of amyloid beta in mouse models of Alzheimer's disease. Nat. Commun. 7, 10759. 10.1038/ncomms10759 26892305 PMC4762893

[B41] Sevigny J. Chiao P. Bussière T. Weinreb P. H. Williams L. Maier M. . (2016). The antibody aducanumab reduces Aβ plaques in Alzheimer's disease. Nature. 537, 50–56. 10.1038/nature19323 27582220

[B42] Shankar G. M. Li S. Mehta T. H. Garcia-Munoz A. Shepardson N. E. Smith I. . (2008). Amyloid-beta protein dimers isolated directly from Alzheimer's brains impair synaptic plasticity and memory. Nature medicine, 14, 837–842. 10.1038/nm1782 18568035 PMC2772133

[B43] Sollvander S. Nikitidou E. Gallasch L. Zysk M. Soderberg L. Sehlin D. . (2018). The A beta protofibril selective antibody mAb158 prevents accumulation of A beta in astrocytes and rescues neurons from A beta-induced cell death. J. Neuroinflammation. 15, 98. 10.1186/s12974-018-1134-4 29592816 PMC5875007

[B44] Swanson C. J. Zhang Y. Dhadda S. Wang J. Kaplow J. Lai R. Y. K. . (2021). A randomized, double-blind, phase 2b proof-of-concept clinical trial in early Alzheimer's disease with lecanemab, an anti-Aβ protofibril antibody. Alzheimers. Res. Ther. 13, 80. 10.1186/s13195-021-00813-8 33865446 PMC8053280

[B45] Tam S. Zhang G. Li L. Elmaarouf A. Skov M. Dolan P. . (2021). PRX012 induces microglia-mediated clearance of pyroglutamate-modified Aβ in Alzheimer's Disease brain tissue. Alzheimer's and Dementia. 17, 1868–1879. 10.1002/alz.057773

[B46] Thomas R. G. Albert M. Petersen R. C. Aisen P. S. (2016). Longitudinal decline in mild-to-moderate Alzheimer's disease: Analyses of placebo data from clinical trials. Alzheimers. Dement. 12, 598–603. 10.1016/j.jalz.2016.01.002 26917500

[B47] Tiwari S. Atluri V. Kaushik A. Yndart A. Nair M. (2019). Alzheimer's disease: pathogenesis, diagnostics, and therapeutics. Int. J. Nanomedicine 14, 5541–5554. 10.2147/IJN.S200490 31410002 PMC6650620

[B48] Tublin J. M. Adelstein J. M. Del Monte F. Combs C. K. Wold L. E. (2019). Getting to the Heart of Alzheimer Disease. Circ. Res. 124, 142–149. 10.1161/CIRCRESAHA.118.313563 30605407 PMC6319653

[B49] Van Dyck C. H. Swanson C. J. Aisen P. Bateman R. J. Chen C. Gee M. . (2023). Lecanemab in early Alzheimer's disease. N. Engl. J. Med. 388, 9–21. 10.1056/NEJMoa2212948 36449413

[B50] Wan X. Wang W. Liu J. Tong T. (2014). Estimating the sample mean and standard deviation from the sample size, median, range and/or interquartile range. BMC Med. Res. Methodol. 14, 135. 10.1186/1471-2288-14-135 25524443 PMC4383202

[B51] Wilson H. Pagano G. Politis M. (2019). Dementia spectrum disorders: lessons learnt from decades with PET research. J. Neural. Transm. (Vienna). 126, 233–251. 10.1007/s00702-019-01975-4 30762136 PMC6449308

