# Supplementary material for: Safety and efficacy of lecanemab for Alzheimer's disease: a systematic review and meta-analysis of randomized clinical trials
Source: Front Aging Neurosci. 2023 May 5;15:1169499. doi: 10.3389/fnagi.2023.1169499 (PMC10196238; doi:10.3389/fnagi.2023.1169499)
Supplement: Supplementary file 1 [file Table_1.DOCX]

Supplementary Material

Safety and efficacy of lecanemab for Alzheimer’s disease: a systematic review and meta-analysis of randomized clinical trials

**Yue Qiao^1^, Yuewei Chi^1^, Qingyuan Zhang^1^, Ying Ma^1*^**

**^1^**Department of Neurology, Shengjing Hospital of China Medical University, Shenyang, China

**^*^** Corresponding author. Address correspondence to: *Ying Ma, PhD*, *Shengjing Hospital of China*

*Medical University, Shenyang, Liaoning, China, 110817,*

*E-mail:*mayingwfd@163.com

Supplementary Table S1. Detailed search strategy in four databases.

| Database | Search strategy |
| --- | --- |
| Pubmed | : (("lecanemab" [Supplementary Concept]) OR (((((lecanemab-irmb) OR (leqembi)) OR (BAN2401)) OR (BAN 2401)) OR (BAN-2401))) AND ((((((((((((((((((((((((((((((((((((Alzheimer Dementia) OR (Alzheimer Dementias)) OR (Dementia, Alzheimer)) OR (Alzheimer's Disease)) OR (Dementia, Senile)) OR (Senile Dementia)) OR (Dementia, Alzheimer Type)) OR (Alzheimer Type Dementia)) OR (Alzheimer-Type Dementia (ATD))) OR (Alzheimer Type Dementia (ATD))) OR (Dementia, Alzheimer-Type (ATD))) OR (Alzheimer Type Senile Dementia)) OR (Primary Senile Degenerative Dementia)) OR (Dementia, Primary Senile Degenerative)) OR (Alzheimer Sclerosis)) OR (Sclerosis, Alzheimer)) OR (Alzheimer Syndrome)) OR (Alzheimer's Diseases)) OR (Alzheimer Diseases)) OR (Alzheimers Diseases)) OR (Senile Dementia, Alzheimer Type)) OR (Acute Confusional Senile Dementia)) OR (Senile Dementia, Acute Confusional)) OR (Dementia, Presenile)) OR (Presenile Dementia)) OR (Alzheimer Disease, Late Onset)) OR (Late Onset Alzheimer Disease)) OR (Alzheimer's Disease, Focal Onset)) OR (Focal Onset Alzheimer's Disease)) OR (Familial Alzheimer Disease (FAD))) OR (Alzheimer Disease, Familial (FAD))) OR (Familial Alzheimer Diseases (FAD))) OR (Alzheimer Disease, Early Onset)) OR (Early Onset Alzheimer Disease)) OR (Presenile Alzheimer Dementia)) OR ("Alzheimer Disease"[Mesh])) |
| Embase^*^ | 1 (lecanemab OR lecanemab-irmb OR leqembi OR BAN2401 OR BAN 2401 OR BAN-2401).af.  2 (Alzheimer's Disease OR Alzheimer Dementia OR Alzheimer Dementias OR Dementia, Alzheimer OR Alzheimer's Disease OR Dementia, Senile OR Senile Dementia OR Dementia, Alzheimer Type OR Alzheimer Type Dementia OR Alzheimer-Type Dementia (ATD) OR Alzheimer Type Dementia (ATD) OR Dementia, Alzheimer-Type (ATD) OR Alzheimer Type Senile Dementia OR Primary Senile Degenerative Dementia OR Dementia, Primary Senile Degenerative OR Alzheimer Sclerosis OR Sclerosis, Alzheimer OR Alzheimer Syndrome OR Alzheimer's Diseases OR Alzheimer Diseases OR Alzheimers Diseases OR Senile Dementia, Alzheimer Type OR Acute Confusional Senile Dementia OR Senile Dementia, Acute Confusional OR Dementia, Presenile OR Presenile Dementia OR Alzheimer Disease, Late Onset OR Late Onset Alzheimer Disease OR Alzheimer's Disease, Focal Onset OR Focal Onset Alzheimer's Disease OR Familial Alzheimer Disease (FAD) OR Alzheimer Disease, Familial (FAD) OR Familial Alzheimer Diseases (FAD) OR Alzheimer Disease, Early Onset OR Early Onset Alzheimer Disease OR Presenile Alzheimer Dementia).af.  3 1 and 2 |
| Web of Science | 1 lecanemab (Topic) OR lecanemab-irmb (Topic) OR leqembi (Topic) OR BAN2401 (Topic) OR BAN 2401 (Topic) OR BAN-2401 (Topic)  2 Alzheimer's Disease (Topic) OR Alzheimer Dementia (Topic) OR Alzheimer Dementias (Topic) OR Dementia, Alzheimer (Topic) OR Alzheimer's Disease (Topic) OR Dementia, Senile (Topic) OR Senile Dementia (Topic) OR Dementia, Alzheimer Type (Topic) OR Alzheimer Type Dementia (Topic) OR Alzheimer-Type Dementia (ATD) (Topic) OR Alzheimer Type Dementia (ATD) (Topic) OR Dementia, Alzheimer-Type (ATD) (Topic) OR Alzheimer Type Senile Dementia (Topic) OR Primary Senile Degenerative Dementia (Topic) OR Dementia, Primary Senile Degenerative (Topic) OR Alzheimer Sclerosis (Topic) OR Sclerosis, Alzheimer (Topic) OR Alzheimer Syndrome (Topic) OR Alzheimer's Diseases (Topic) OR Alzheimer Diseases (Topic) OR Alzheimers Diseases (Topic) OR Senile Dementia, Alzheimer Type (Topic) OR Acute Confusional Senile Dementia (Topic) OR Senile Dementia, Acute Confusional (Topic) OR Dementia, Presenile (Topic) OR Presenile Dementia (Topic) OR Alzheimer Disease, Late Onset (Topic) OR Late Onset Alzheimer Disease (Topic) OR Alzheimer's Disease, Focal Onset (Topic) OR Focal Onset Alzheimer's Disease (Topic) OR Familial Alzheimer Disease (FAD) (Topic) OR Alzheimer Disease, Familial (FAD) (Topic) OR Familial Alzheimer Diseases (FAD) (Topic) OR Alzheimer Disease, Early Onset (Topic) OR Early Onset Alzheimer Disease (Topic) OR Presenile Alzheimer Dementia Topic  3 #1 AND #2 |
| Cochrane | #1 (lecanemab):ti,ab,kw OR (lecanemab-irmb):ti,ab,kw OR (leqembi):ti,ab,kw OR (BAN2401):ti,ab,kw OR (BAN 2401):ti,ab,kw  #2 (BAN-2401):ti,ab,kw  #3 MeSH descriptor: [Alzheimer Disease] explode all trees  #4 (Alzheimer's Disease):ti,ab,kw OR (Alzheimer Dementia):ti,ab,kw OR (Alzheimer Dementias):ti,ab,kw OR (Dementia, Alzheimer):ti,ab,kw OR (Alzheimer's Disease):ti,ab,kw  #5 (Dementia, Senile):ti,ab,kw OR (Senile Dementia):ti,ab,kw OR (Dementia, Alzheimer Type):ti,ab,kw OR (Alzheimer Type Dementia):ti,ab,kw OR (Alzheimer-Type Dementia (ATD)):ti,ab,kw  #6 (Alzheimer Type Dementia (ATD)):ti,ab,kw OR (Dementia, Alzheimer-Type (ATD)):ti,ab,kw OR (Alzheimer Type Senile Dementia):ti,ab,kw OR (Primary Senile Degenerative Dementia):ti,ab,kw OR (Dementia, Primary Senile Degenerative):ti,ab,kw  #7 (Alzheimer Sclerosis):ti,ab,kw OR (Sclerosis, Alzheimer):ti,ab,kw OR (Alzheimer Syndrome):ti,ab,kw OR (Alzheimer's Diseases):ti,ab,kw OR (Alzheimer Diseases):ti,ab,kw  #8 (Alzheimers Diseases):ti,ab,kw OR (Senile Dementia, Alzheimer Type):ti,ab,kw OR (Acute Confusional Senile Dementia):ti,ab,kw OR (Senile Dementia, Acute Confusional):ti,ab,kw OR (Dementia, Presenile):ti,ab,kw  #9 (Presenile Dementia):ti,ab,kw OR (Alzheimer Disease, Late Onset):ti,ab,kw OR (Late Onset Alzheimer Disease):ti,ab,kw OR (Alzheimer's Disease, Focal Onset):ti,ab,kw OR (Focal Onset Alzheimer's Disease):ti,ab,kw  #10 (Familial Alzheimer Disease (FAD)):ti,ab,kw OR (Alzheimer Disease, Familial (FAD)):ti,ab,kw OR (Familial Alzheimer Diseases (FAD)):ti,ab,kw OR (Alzheimer Disease, Early Onset):ti,ab,kw OR (Early Onset Alzheimer Disease):ti,ab,kw  #11 (Presenile Alzheimer Dementia):ti,ab,kw  #12 #1OR#2  #13 #3OR#4OR#5OR#6OR#7OR#8OR#9OR#10OR#11  #14 #12AND#13 |
| ^*^ We retrieved articles from Embase via Ovid (https://ovidsp.ovid.com/). | |
